# Supplementary material for: Ferroelectric nematic liquid crystals: temperature-tunable electrocaloric fluids with large strength at low fields
Source: Npj Therm Sci Eng. 2026 Oct 7;1(1):18. doi: 10.1038/s44435-026-00019-0 (PMC13671268; doi:10.1038/s44435-026-00019-0)
Supplement: Supplementary file 1 [file 44435_2026_19_MOESM1_ESM.pdf]

Ferroelectric nematic liquid crystals: temperature-tunable electrocaloric fluids with large strength at low fields – Supplementary Information

Supplementary Note I: Entropy change in cuvettes

Data for the entropy change obtained via the indirect Maxwell method is shown in Figure S1.

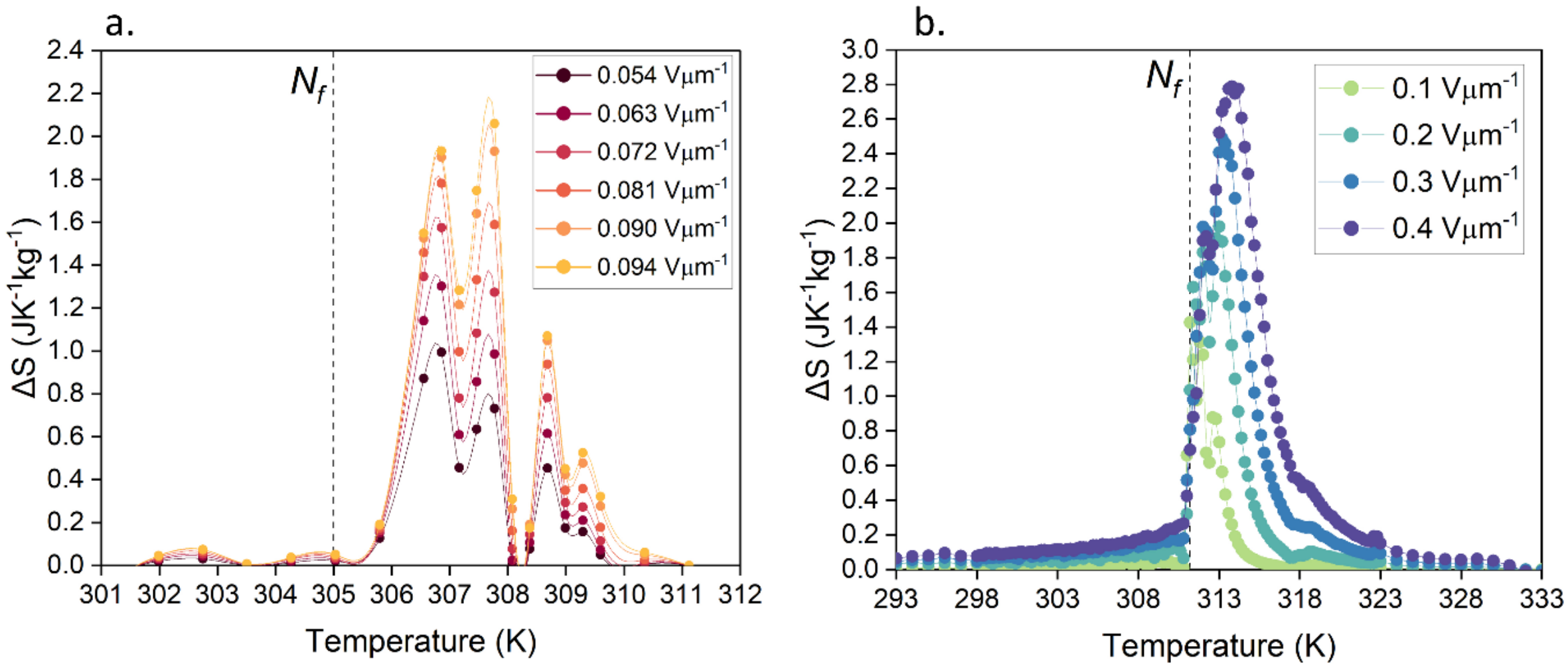

FIG. S1. Measurements of entropy change, ΔS, as function of temperature in both materials. (a) Entropy change in FNLC-919 across a temperature range of 301-312 K, at six applied electric fields (0.054-0.094 V μm^{-1}). (b) Entropy change in fnlc-346 across a temperature range of 293-333 K, at four applied electric fields (0.1-0.4 V μm^{-1}). In both graphs, the ferroelectric nematic transition is indicated by a dashed line and labelled.

Supplementary Note II: Additional indirect measurements

Glass cells with ITO (electrode) coating on the inside of the glass substrates are commonly used in measurements on liquid crystals. The glass substrates have a thickness of ~1 mm. The gap between electrodes (referred to as cell gap or the LC thickness layer) can vary in thickness, though the thickness for cells shown in Figure S1 was 6 μm. Such a thin LC layer allows the application of a much larger electric field than can be achieved in the cuvettes (which have a gap of 1 mm), and consequently we can obtain larger values for the electrocaloric temperature change. $P(E)$ data taken in such glass cells is shown in Figure S1.

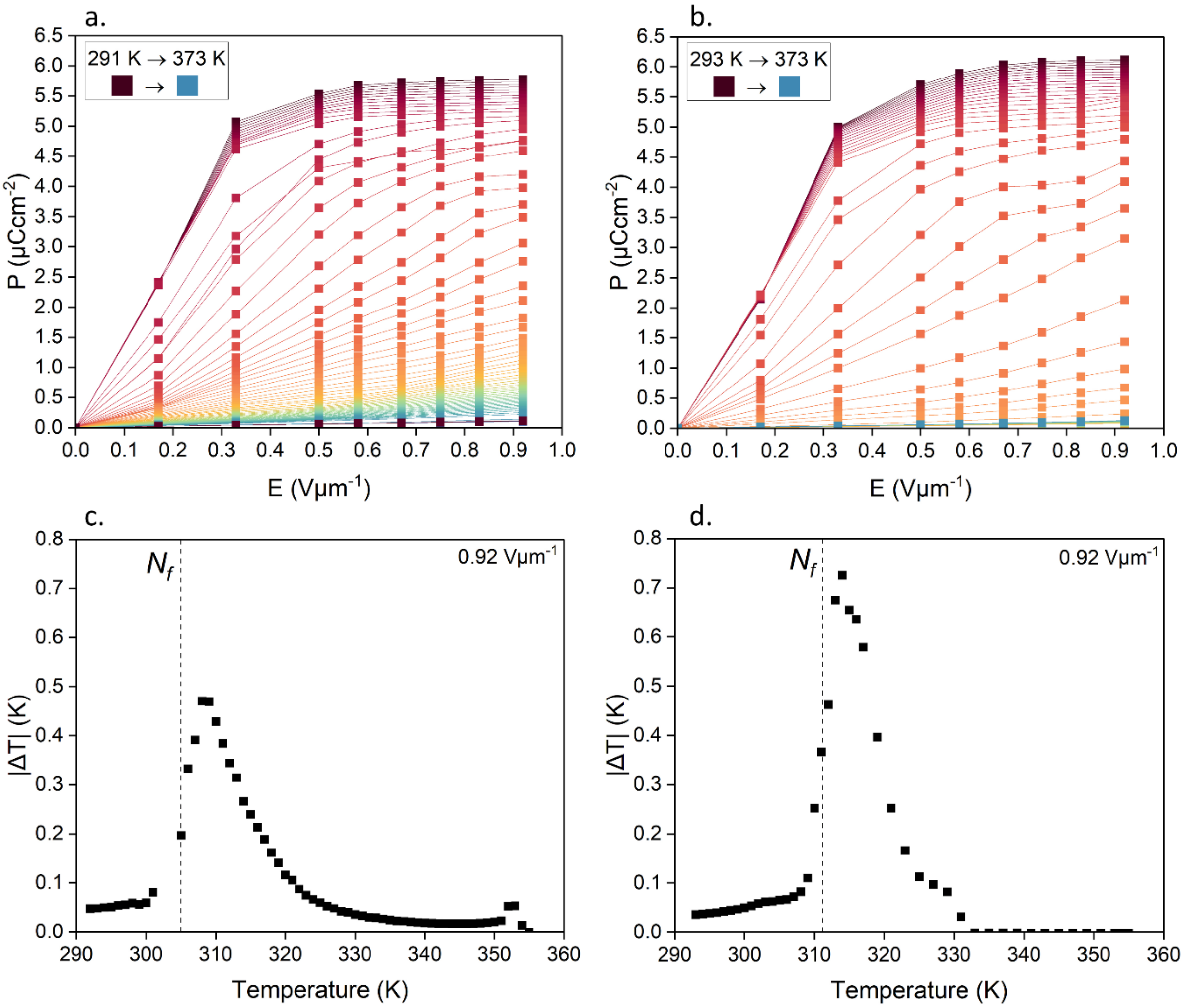

FIG. S2. Indirect EC measurements in standard liquid crystal cells. (a) P(E) data for FNLC-919 for a range of temperatures. (b) P(E) data for FNLC-346 for a range of temperatures. These data were used to find the largest electrocaloric temperature change via the indirect method, shown in (c) for FNLC-919 and (d) for FNLC-346.

At applied field E = 0.92 Vμm^{-1}, the highest obtained temperature change is $|\Delta T|$ = 0.47 K at set temperature T = 308 K for FNLC-919, and $|\Delta T|$ = 0.73 K at set temperature T = 314 K for FNLC-346. As expected, the electrocaloric effect is strongest just above the ferroelectric phase transition for both materials. Additionally, these values are in good agreement with the temperature change values discussed in the manuscript, which were obtained in cuvettes.

We note the difference in P(E) curves for cuvettes and cells with respect to the magnitude of applied field necessary to reach the maximum value of polarisation, P_{max}. In FNLC-919, P_{max} is reached at E = 0.05 Vμm^{-1} in cuvettes and at E = 0.5 Vμm^{-1} in cells – a difference of one order of magnitude. Similarly in FNLC-346, P_{max} requires E = 0.1 Vμm^{-1} in cuvettes but E = 0.7 Vμm^{-1} in cells. As briefly mentioned in the main text, and discussed further in Ref. [26] (in manuscript), polarisation switching in ferroelectric nematic liquid crystals depends highly

on surface interactions and not so much on the cell gap thickness (even when the difference in cell gap thickness between the cells (6μm) and cuvettes (1mm) is significant). Indeed, the aluminium electrodes in the cuvettes appear to have a more favourable interaction with the liquid crystal than the ITO coating in the conventional device, allowing for the use of much smaller electric fields in achieving similar electrocaloric temperature change.

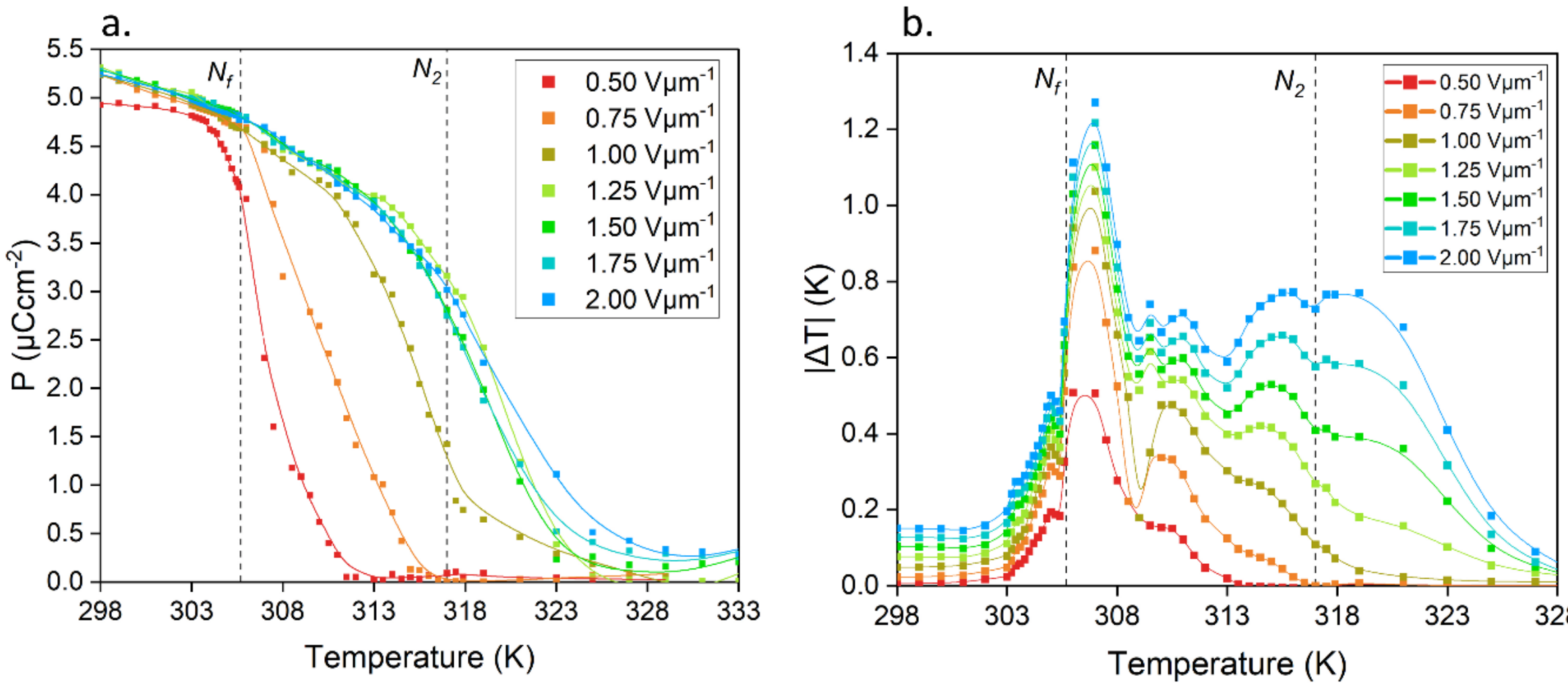

FIG. S3. Indirect electrocaloric measurements of FNLC-919 in a cell (thickness ~ 5 μm). (a) Polarisation measurements taken at 63Hz, across both the ferroelectric nematic and intermediate phases (labelled) at a range of applied fields (0.5-2.0 V μm^{-1}). The associated electrocaloric temperature change is shown in (b).

Measurements in devices with small electrode gap allow the application of higher fields to the FNLC so that the potential of the electrocaloric effect in liquid crystals may be explored more fully. As can be seen in Figure S2, fields up to E = 2 Vμm^{-1} could be applied to the device, achieving a temperature change of $|\Delta T|$ = 1.2 K, the highest we have reported so far. The second, broader peak in the temperature change data in S2(b) is likely not real but rather due to the broader transition causing changes in the polarisation differential that would not be there in a sharp first-order transition. Such a large second peak is not observed in the indirect data in cuvettes.

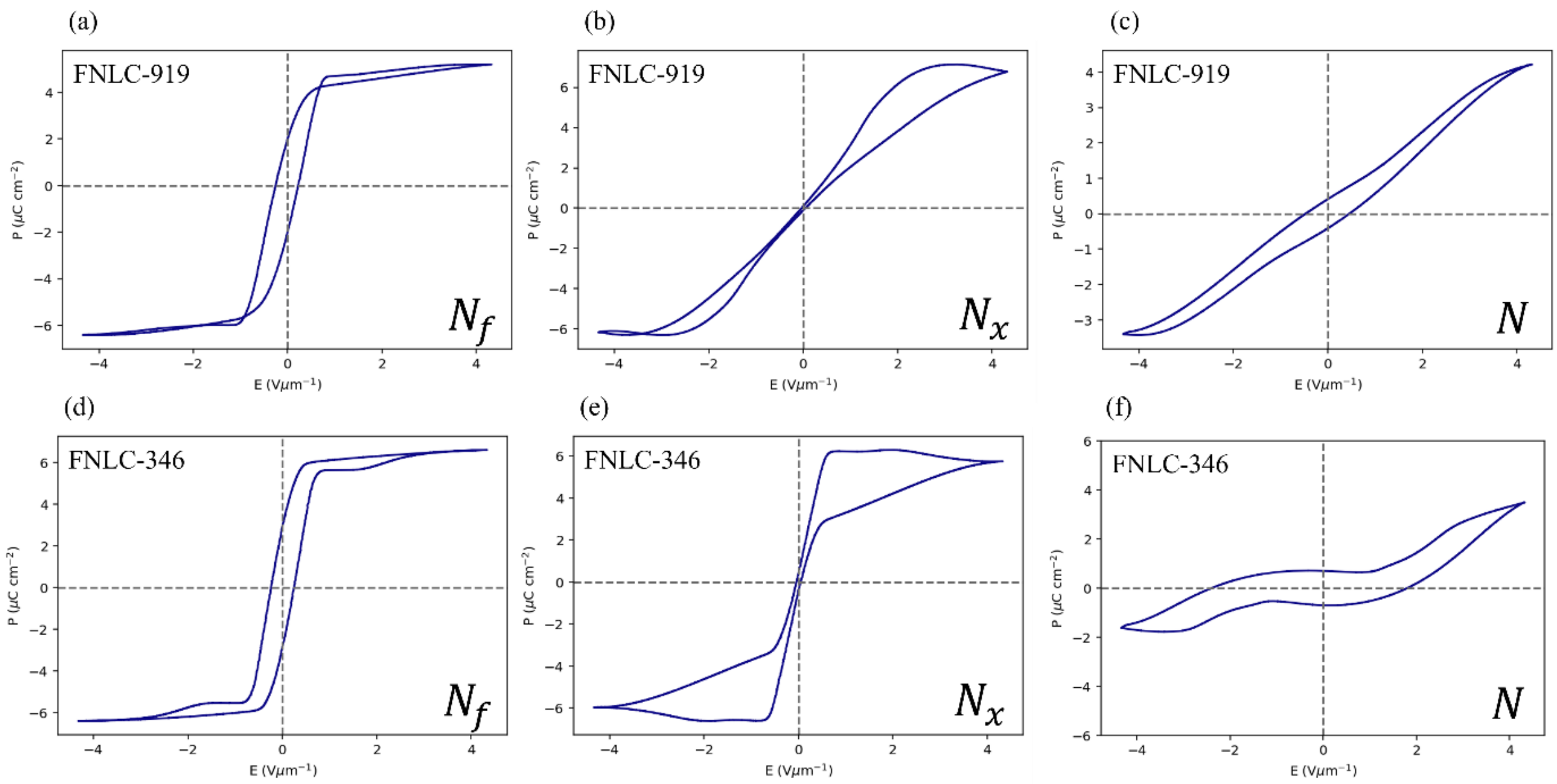

FIG. S4. Representative P-E hysteresis loops in the N_f, N_x, and N phases for both materials. (a-c) Hysteresis loops in FNLC-919. (d-f) Hysteresis loops in FNLC-346.

Some representative hysteresis loops have been included here for both materials. These were obtained by numerically integrating the current output data from applying the electric field across 4μm thick cells with no alignment layer.

Supplementary Note III: Methodology details

The cuvettes used in direct electrocaloric measurements in this work have a gap between the electrodes of 1 mm. The electrodes are aluminium, with a thickness of 0.55 mm and inner surface area of approximately 1.1 ± 0.1 mm^2. On average, about 200mg of material is sufficient to fill a cuvette using the full electrode area.

The thermocouple used for direct measurements is a PFA insulated type K thermocouple (RS Components Ltd.). The wires have diameters of 0.076 mm, whereas the welded tip has a diameter of 0.419 ± 0.002 mm.

Both indirect and direct measurements were repeated in two separate experimental settings: at the University of Leeds and repeated by Merck Electronics KGaA. The methodology employed was the same in both settings, with some differences in type/model of equipment and level of automation. This was done as a way to verify the results obtained by both parties, especially in the case of direct measurements.

Supplementary Note IV: COP in FNLC-919

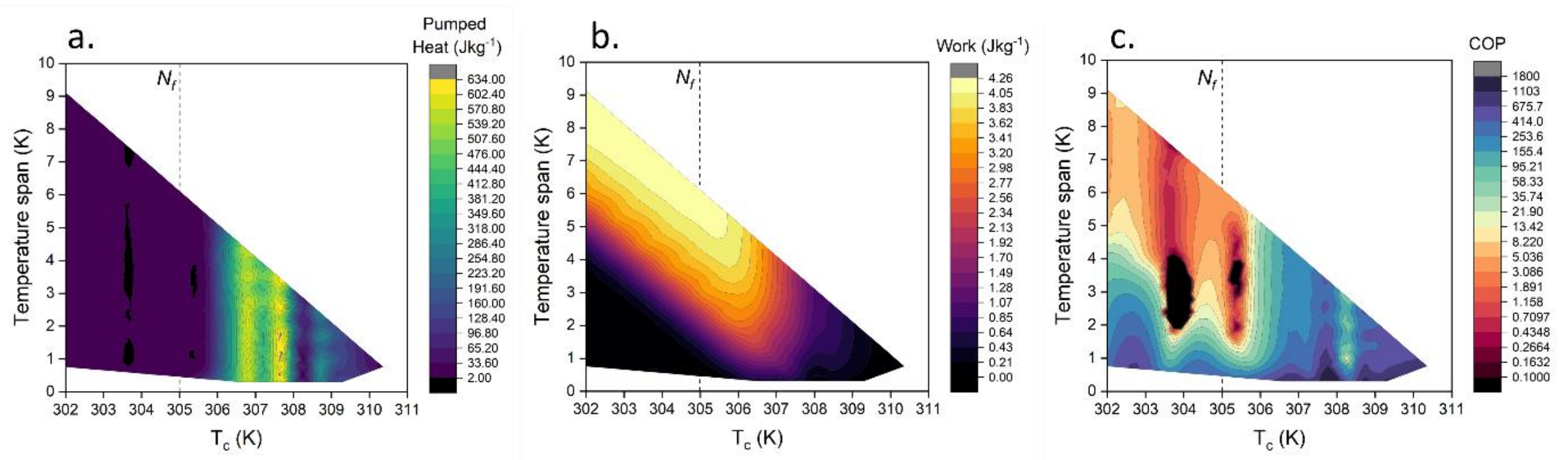

FIG. S5. Coefficient of performance data for FNLC-919. (a) Colour map of the pumped heat, Q. (b) Colour map of the work, W. (c) COP values for FNLC-919 at the cold end temperature T_c, calculated using an Ericsson cycle as shown for FNLC-346 in the manuscript. In all three figures, the ferroelectric nematic transition is indicated with a dashed line.

A calculation was done for COP values of FNLC-919 based off the entropy change measurements shown previously in Figure S1. Near the ferroelectric transition, we see COP ~ 8-12 for temperature spans of 3-4 K. There are, however, black spots in the graph corresponding to COP ~ 0 which can be attributed to the multiple peaks present in the entropy change data. The corresponding calculation for FNLC-346 is shown in the manuscript.

Supplementary Note V: DSC

Here we provide the full range of the DSC curves on cooling for both mixtures, FNLC-919 and FNLC-346. The tested samples are 14.2 mg (FNLC-919) and 21.5 mg (FNLC-346).

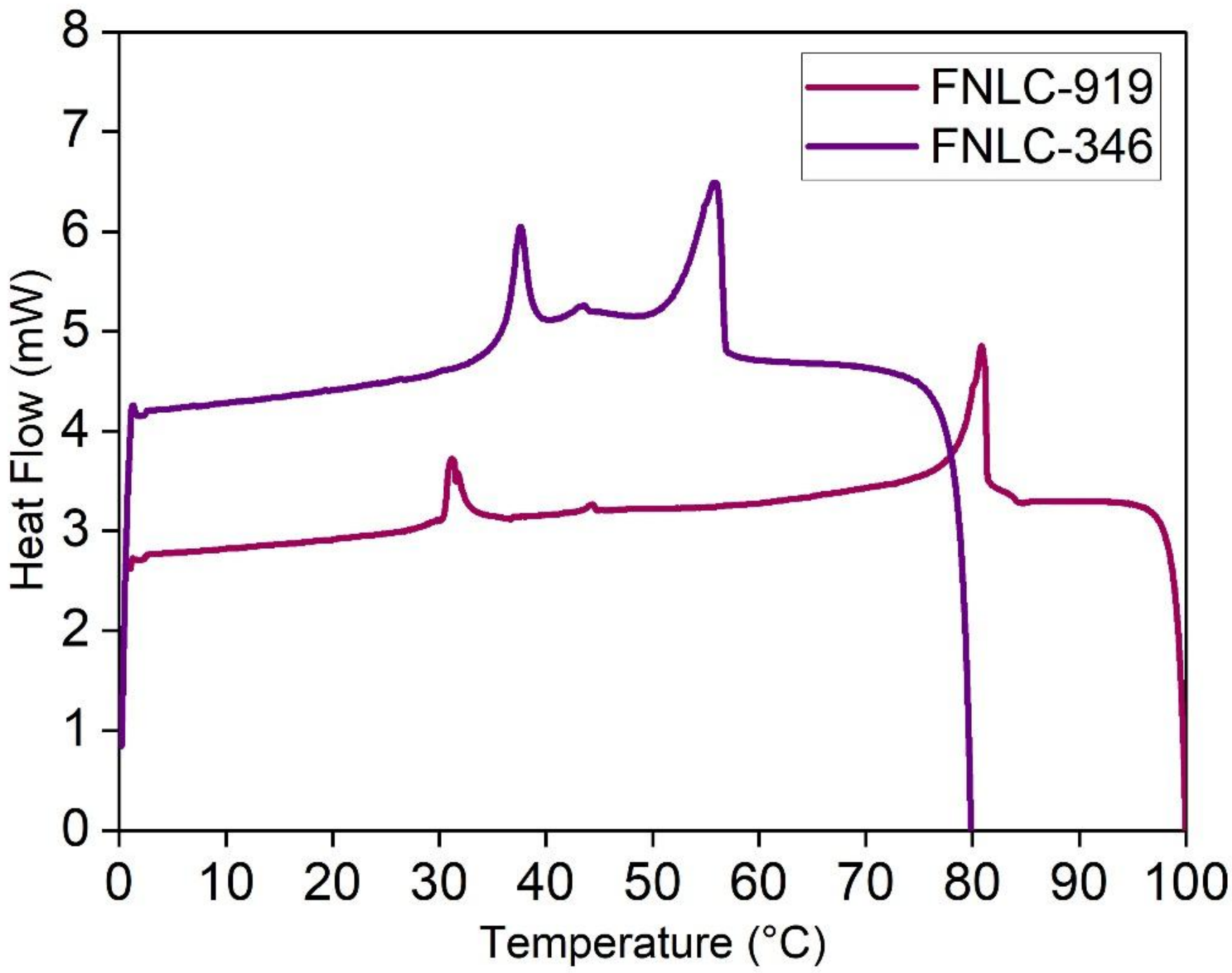

FIG. S6. Heat flow plots from DSC for FNLC-919 and FNLC-346 across the full temperature range.

Supplementary Note VI: Polarised Optical Microscopy Images

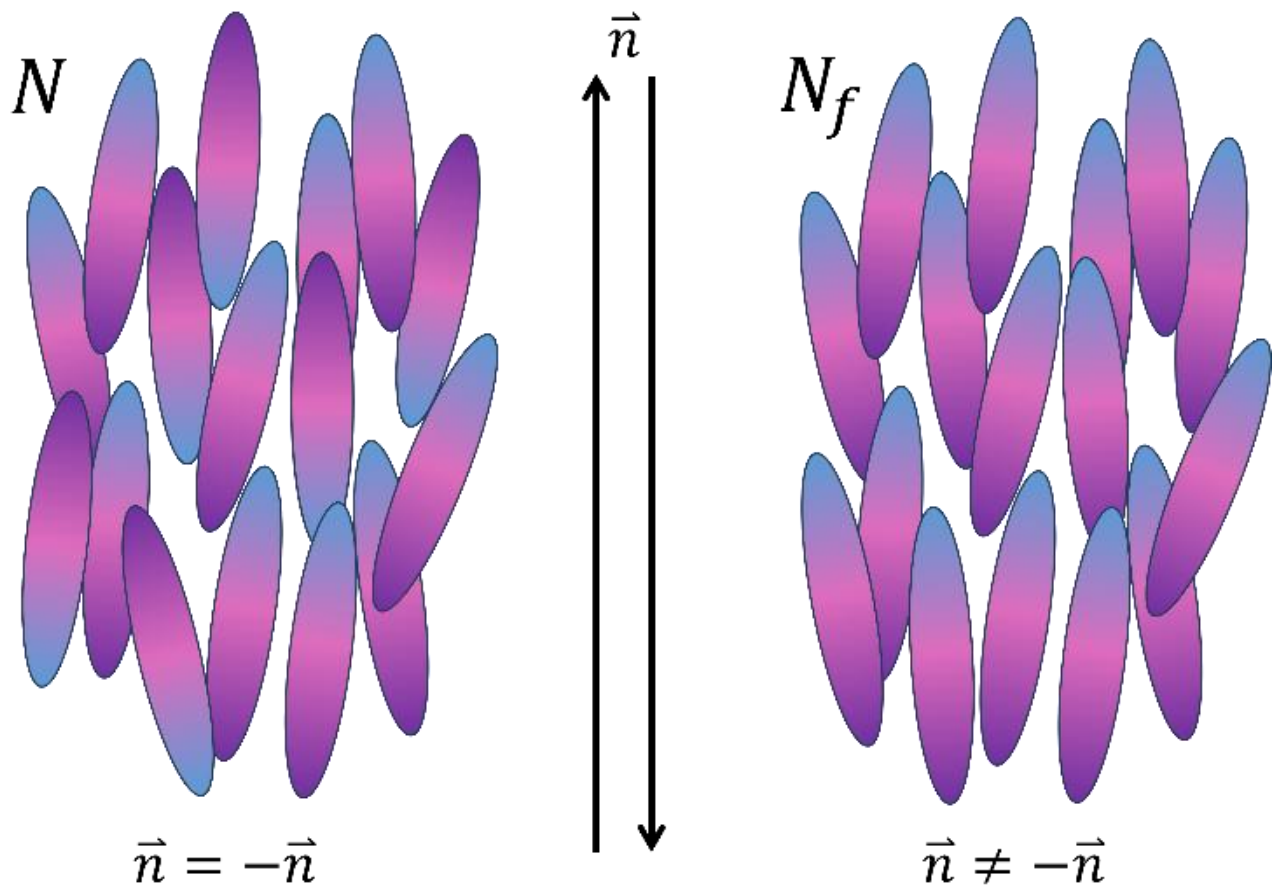

FIG. S7. Illustrative schematic showing the difference between the nematic (left) and the ferroelectric nematic (right) phases. The arrows indicate the director, a unit vector which defines the average orientation of the molecular long axes which is a characteristic of nematic phases that possess orientational order. The permanent dipole on the molecules is indicated by the colouring, so that blue and purple ends of the molecules refer to positive and negative dipole moments. The N phase has inversion symmetry as there is no net orientation of the molecular dipoles. However, in the N_f phase, the molecular dipoles are ordered, resulting in a macroscopically polar (ferroelectric) phase.

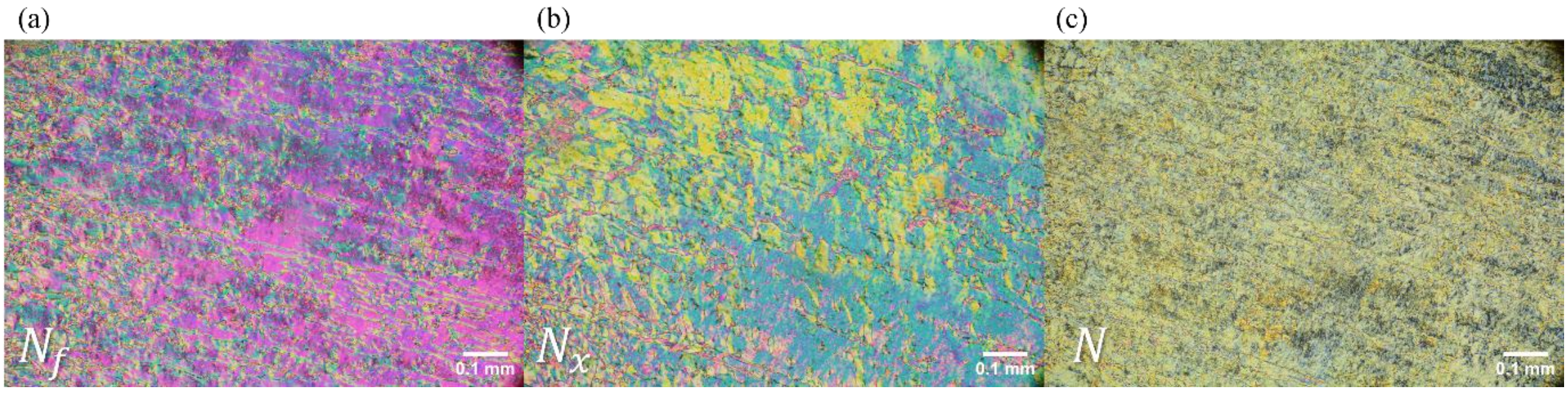

FIG. S8. FNLC-346 in a 4 μm thick cell with no alignment. (a) Image taken in N_f phase. (b) Image taken in N_x phase. (c) Image taken in N phase. A scale bar is given in the lower right corner of each image. No field is applied.

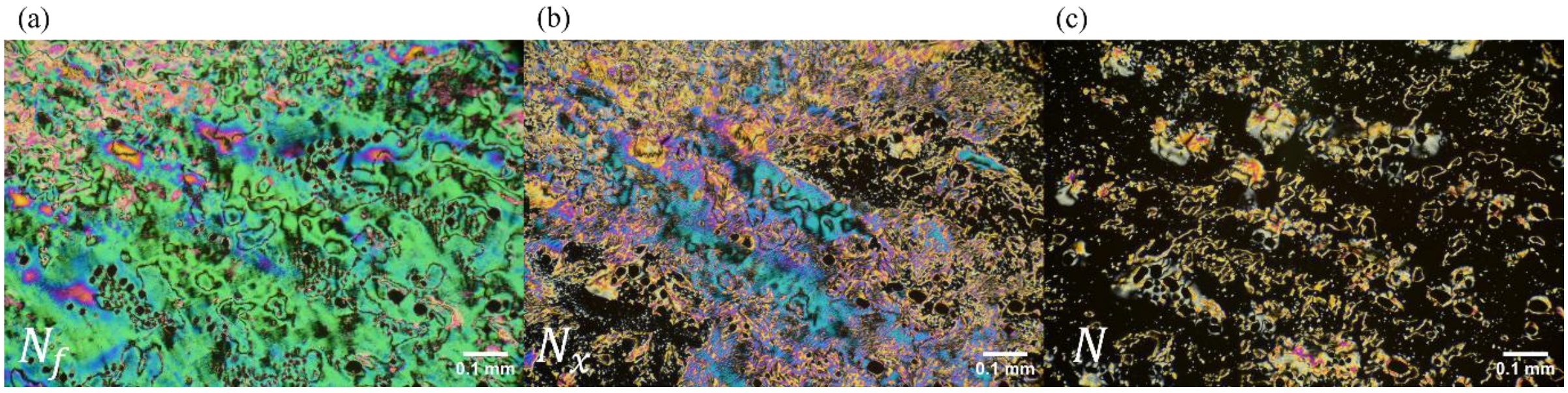

FIG. S9. FNLC-919 in a 4 μm thick cell with no alignment. (a) Image taken in N_f phase. (b) Image taken in N_x phase. (c) Image taken in N phase. A scale bar is given in the lower right corner of each image. No field is applied.

Images of each material under polarised light are included here to aid with the understanding of the materials, though we note that the electrocaloric measurements are done in cuvettes with an electrode gap of 1mm, which is significantly larger than the thickness of the cells used in these pictures. Polarised microscopy images could not be obtained in the thick cuvette samples.

It can be seen that both materials exhibit interesting patterns when there is no alignment in the cells. For the ferroelectric phase, the polarity means that the director cannot be perpendicular to the substrate, so energy is minimized by forming the patterns and birefringent colours seen in Fig. S8(a) and S9(a). Similarly in Fig. S8(b) and S9(b) in the antiferroelectric phase, the patterns largely remain, likely due to anchoring to the glass surfaces of the substrates, but the birefringent colours change as the director reorients during the phase change. In Fig. S9(b) one can even observe black regions growing, which is most likely regions where the material is homeotropically aligned (director is perpendicular to the substrate) as the macroscopic domain polarity is now broken. The dark regions grow and overtake the image as we transition into the nematic phase, in Fig. S9(c). This is not as apparent in FNLC-346, which has phase transitions much closer together in temperature than FNLC-919 and it is possible the ordering in the ferroelectric and antiferroelectric phases still affects the ordering in the nematic phase (Fig. S8(c)). Although this is not shown here, we note that in the N_x and N phases, homeotropic alignment of the material can be achieved via the application of electric field even in these unaligned cells. Such alignment cannot be achieved here in the N_f phase.

More polarized optical microscopy images and discussion on ferroelectric nematic liquid crystals can be found in a number of publications, such as for example: [Sebastián, N., Čopič, M., & Mertelj, A. (2022). Ferroelectric nematic liquid-crystalline phases. Physical Review E, 106(2), 021001.] and [Lavrentovich, M.O., Kumari, P. & Lavrentovich, O.D. Twist, splay, and uniform domains in ferroelectric nematic liquid crystals. Nat Commun 16, 6516 (2025).]
